# Autologous fat grafting to the paravertebral space seems to prevent the postherpetic neuralgia—A single‐arm pilot study

**DOI:** 10.1002/brb3.2918

**Published:** 2023-03-14

**Authors:** Xiujuan Li, Ran Tao, Xiaoyan Meng, Li Sun, Han Wang, Yuanyuan Sun, Hongda Bi, Yuanchang Xiong

**Affiliations:** ^1^ Department of Anesthesiology and Perioperative Medicine, Shanghai Fourth People's Hospital, School of Medicine Tongji University Shanghai China; ^2^ Department of Anesthesiology and Pain Center, Shanghai Changhai Hospital Naval Medical University Shanghai China; ^3^ Department of Plastic Surgery, Shanghai Changhai Hospital Naval Medical University Shanghai China; ^4^ Department of Critical Care Medicine, Eastern Hepatobiliary Surgery Hospital Navel Medical University Shanghai China

**Keywords:** autologous fat grafting, herpes zoster, paravertebral space, pilot study, postherpetic neuralgia

## Abstract

**Introduction:**

Postherpetic neuralgia (PHN) is one of the most common complications of Herpes zoster (HZ), yet the mechanism and the treatment for PHN remains elusive. We first performed this feasibility study to verify the safety and efficiency of autologous fat grafting into the paravertebral space in early HZ to prevent PHN.

**Methods:**

Patients suffering from HZ with a rash in chest, back, or abdomen were arranged for autologous fat grafting to the paravertebral space. The primary endpoint was the incidence of PHN, which was defined as persistence pain in the affected dermal area in 12 weeks after fat grafting. Secondary endpoints including patient‐reported changes in pain intensity, assessed pain threshold and the quality of life during follow‐ups.

**Results:**

Eight patients accept the intervention and completed all follow‐ups. Most patients report immediate pain relief after injection, one patient has a mild to moderate dizzy symptom after injection. No other short‐ or long‐term adverse events occurred. For primary outcome, all patients have a timely reduced pain intensity, with no PHN events occurred, as all patients report pain intensity ≤3 in the VAS scale in 3 months after treatment. For electrical pain threshold, we identify that fat grafting differentially increases sensation and pain threshold in HZ area and healthy skin of patients. Besides, our results indicate significant improvement in patients’ life quality decrease in analgesic consumption.

**Discussion:**

Autologous fat transplantation to the paravertebral space is a safe and feasible technique in preventing PHN from HZ in a rash. Further randomized controlled trial to investigate the actual long‐term benefice of autologous fat grafting to the paravertebral space in preventing PHN is needed.

**Trial registration:**

ChiCTR, (ChiCTR1900025416); registered August 26, 2019.

## Graphical abstract

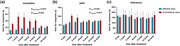

## 1 INTRODUCTION

Herpes zoster (HZ) is a comment disease with distinctive clinical conditions, it is caused after varicella‐zoster virus infection. According to epidemiological investigation, one in four people might suffer from HZ during their lifetime, and for individuals aged over 50, the incidence for HZ increased to almost 50% (David, 2012). The predominant symptom of HZ is painful dermatomal vesicular eruption of blisters and allodynia in the lesion area, which significantly reduced the life quality and increased medical cost for patients.

Postherpetic neuralgia (PHN) is one of the most common complications of HZ, PHN is defined as pain that persists for 3 months or more after the resolution of the initial rash. The risk of PHN is reported between 5% and 30% after the break out of HZ. Advanced age is one of the risk factors for developing PHN, as about 12.5% of HZ patients over 50 years old can develop PHN, and the increase in risk varies from 1.22 to 3.11 for every 10 years of age (Hadley et al., 2016).

Numerous researchers have investigated the potential mechanism for the residual pain of PHN. Theories including peripheral sensitization, central sensitization and peripheral neuroinflammatory responses have been identified, yet the pathogenesis of PHN remains unclear (Hoon, 2015). However, the management for PHN remains challenging, with currently no reliable treatment or internationally accepted guideline for preventing or treating PHN.

One approach to prevent PHN could be autologous fat grafting, a widely used technique in cosmetic surgery and has proven to be a safe procedure in the past 30 years (Bucky & Percec, 2008; Simonacci et al., 2017). In recent years, due attention has been attached on the application of autologous fat grafting in management of neuropathic pains, such as scar related pain, pain after breast surgery and neuralgia after burn (Caviggioli et al., 2011; Huang et al., 2014, 2015). The mechanism for this therapy to reduce chronic pain is uncertain, animal studies infer that adipokines and mesenchymal stem cells in the adipose tissue may have an anti‐inflammatory effect. Sollie et al. (2020) first reported a potential benefice of autologous fat grafting to the dermal area in alleviating PHN. Based on comprehensive analysis of the pathogenesis of PHN and related studies, we hypothesized that autologous fat injection around spinal nerve roots may have a preventive effect for chest‐back PHN. Therefore, randomized clinical trials are need to verify this hypothesis, and we first performed this feasibility study to verify the safety and efficiency of autologous fat grafting into the paravertebral space in early HZ to prevent PHN.

## 2 METHODS

This open‐label pilot study was undertaken in the Pian Center of Shanghai Changhai Hospital, China, from February 1, 2020 to January 30, 2022. The protocol is in accordance with the Standard Protocol Items: Recommendations for Interventional Trials (SPIRIT) statement. This trial was prospectively registered at Chictr.org.cn with ID number ChiCTR1900025416. All participants provided written consent, and approval for the study was obtained from the Ethics Committee of Changhai Hospital (CHEC2019‐113).

### 2.1 Study design

This is a prospective, single‐arm, and single‐center pilot study to investigate autologous fat grafting to the thoracic paravertebral space for elders with herpetic neuralgia in the rash phase, the main object of this trail is to investigate the feasibility and safety of this treatment in preventing PHN. Participants were all new diagnosed HZ patients visiting our outpatient department for significant pain syndrome. All procedures were performed at the Department of Plastic Surgery, Shanghai Changhai Hospital. The eligibility criteria including (1) age ranged from 50 to 80 years old; (2) BMI ranged from 18 to 28 kg/m^2^; (3) new diagnosed HZ with time of rash less than 1 month and a significant pain syndrome (the presence of pain minimum 4 days a week with an intensity of > 3 on a visual analog scale); (4) chest‐back HZ with lesions from T1 to T12; (5) patients able to read and write in Chinese. And the excluding criteria were as follows: (1) severe coagulation disorders or having anticoagulants in the last week; (2) infection at the paraspinal injection site; (3) potential allergic to any agents or device during the fat‐grafting treatment; (4) severe immunosuppression, such as AIDS, malignant tumors, etc.; (5) combined with diabetes or other serious organ dysfunction; (6) refuse for the treatment or without written consent.

### 2.2 Interventions

All included patients were treated with autologous fat grafting to the thoracic paravertebral space with hyperalgesia and allodynia in related region. All procedures were performed by a skilled plastic surgeon. Before harvest, the region was injected by a tumescent solution (0.5% lidocaine + 0.0005% adrenaline). The fat was harvested by a syringe with low negative pressure (0.75–1.0 atmosphere) and blunt tip cannula (2.1–2.4 mm) through a stab incision on the abdomen or thigh. The harvested fat was sedimented for 10 min and then extra tumescent solution was decanted.

After liposuction, patients were transited to the prone position. Then after sterilization, landmarks for HZ affected paravertebral space were identified under transversal ultrasound scanning. Using a 22‐G spinal needle, a total volume of 10–15 mL of harvested fat was injected at once. Epidural spread of the injection was certified by movement of the parietal pleura line under the ultrasound image.

After the interventions, patients were monitored in the recovery room for 2 h. Pain score in 15 min and in next 3 days were assessed and recorded by an individual physician who was not aware of the study protocol.

### 2.3 Electoral pain threshold assessment

In this trial, we assessed the pain threshold based on patients’ response to a gradual increased electrical stimulus (0.1 mA, pulse width 0.3 ms, impulse frequency 50 Hz) using a pain vision detection device (PS‐2100, Nipro Corporation, Osaka, Japan). During each assessment, a 1‐inch square electrode was placed either in the centered of the HZ skin or at the same area of contralateral skin. With the intensity of electrical stimulation increase, patients were asked to click a time‐count device. Three time point was measured based on previous studies (Shengai et al., 2013): the sensation time, time to feel the pain, and the time to an extent of unbearable pain. The electrical stimuli would stop in the third time click. All measures were assessed for 3 times to calculate a mean value. A physician who was blind to this study design was responsible for all measures and safety of patients.

### 2.4 Data collection and outcomes

Baseline data collected included demographic information; pain level (as perceived by the patient using visual analog scale, VAS, a 10‐cm unmarked line, with anchors: 0 = no pain and 10 cm = worst pain imaginable); current medications; smoking status; and pain threshold of the patient's affected dermal area as well as counterpart skin at healthy side.

After intervention, in 1, 2, 4, 8, 12, 24 weeks after autologous fat injection, participants were asked to having the follow‐up visit at our department. At each visit, information were recorded including: the level of pain at the HZ site, the total analgesic consumption, the times of complete resolution of pain (from the date of paravertebral block until complete disappearance of herpetic pain) and skin eruption (identified by drop of last crust), and pain threshold. The quality of life was measured using the Short‐Form 36 (SF‐36) and the quality of neuropathic pain was recorded according to patients’ self‐report. A 5‐point Likert scale measuring treatment satisfaction (1 = very unsatisfied, 2 = unsatisfied, 3 = neither satisfied nor unsatisfied, 4 = satisfied and 5 = very satisfied) was also recorded. The primary outcome is the incidence of herpetic neuralgia in 3 months after this treatment, as this is determined according to the definition of PHN (Chan, 2016).

All patients’ reported outcomes were collected based on standardized case report form filled out by patients at baseline and at follow‐ups. A pain physician who was blinded to the study protocol, was responsible for the follow‐up and the data collection.

### 2.5 Sample size and statistical methods

This is a hypothesis‐generating feasibility study; the collected data and information are supposed to identify the safety and treatment responses, and to calculate a preliminary sample size for a randomized controlled trial. We chose to include a total of eight patients in this trial. Data were analyzed using the SPSS version 25 (IBM, Inc., Chicago, IL). One‐way ANOVA is used for analysis of VAS score in different time points, and a two‐way ANOVA is applied for analysis of VAS score with variables of time and skin position. A *p* value < .05 was considered statistically significant.

## 3 RESULTS

Eight patients accept the intervention and complete all follow‐ups. All of them are female, with a mean age of 63.7 years (range from 50 to 76 years), while seven of them have a high school degree. All patients are suffered from chest, back or abdominal pain due to emerging herpes zoster, the mean duration for pain is 19.8 days (range from 10 to 27 days), with a rash appeared in about 1–5 days after pain. All patients have accepted or are accepting standard antivirus and analgesic medication. The affected spinal segment including T2–T12, demographic characters for all patients are presented in Table 1.

**TABLE 1:** Demographic characteristics for all participants

ID	Gender	Age	Height (cm)	Weight (kg)	Education	Medical history	Chief compliant	Rash	Segment	Concomitant symptoms
1	Female	59	158	67	High school	Hypertension	Left chest and back pain, 19 days	16 days	T6, T7	Sleep disorder
2	Female	50	165	80	College	Smoking/drinking	Left chest and back pain, 10 days	8 days	T4, T5	Sleep disorder
3	Female	68	150	47	High school	Hyperlipemia	Left chest and back pain, 27 days	27 days, healed	T6‐T8	Sleep disorder
4	Female	64	160	63	High school	None	Left lower back and abdominal pain, 22 days	17 days, healed	T9‐T11	Sleep disorder
5	Female	62	160	52	High school	None	Left back and abdominal pain, 15 days	11 days	T4‐T6	Anxiety, sleep disorder
6	Female	69	157	58	High school	None	Right abdominal pain, 23 days	20 days, healed	T11, T12	Sleep disorder
7	Female	65	156	51	High school	Hypertension	Right chest and back pain, 20 days	15 days	T2‐T4	Anxiety, sleep disorder
8	Female	76	151	55	High school	None	Left back and abdominal pain, 23 days	20 days, healed	T10‐T12	Anxiety, sleep disorder

Four patients have fat harvested from the lower abdomen, while remaining of them had fat harvested from the thighs. The amount of harvested fat ranges from 10–15 mL, according to patients’ physique. Liposuction and injection associated factors are presented in Table 2. Since the solution of the harvested fat also contains lidocaine with a concentration of 0.05%–0.1%, patients accepted precise injection are supposed to have an immediate pain relief. However, one patient report only 10% of pain relief immediately after injection. This pain relief effect lasts for 6–72 h accordingly. One patient had a mild to moderate dizzy symptom after injection, and recovers after 2 h rest.

**TABLE 2:** Treatment associated variables

ID	Areas for liposuction	Amount of harvested fat (mL)	Injection site	Immediate pain relief	Time for pain relief (h)	Adverse events
1	Right lower quadrant abdomen	10	T6	50% or more	48	None
2	Right lower quadrant abdomen	10	T4	Nearly complete	12	None
3	Right lower quadrant abdomen	15	T6	30%	48	None
4	Right thigh	15	T10	Nearly complete	72	None
5	Right thigh	15	T5	50% or more	48	Dizzy after injection
6	Left thigh	10	T11	50% or more	6–12	None
7	Left lower quadrant abdomen	15	T3	10%	12	None
8	Right thigh	15	T11	50% or more	48	None

**FIGURE 1:**
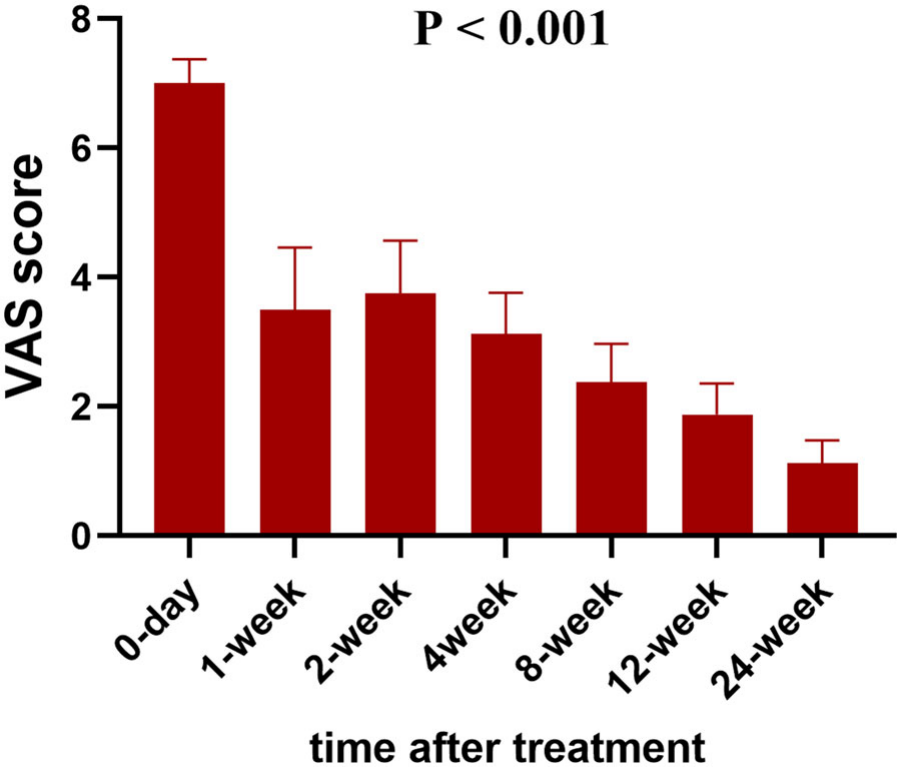
The degree of average pain after autologous fat grafting to the paravertebral space.

**FIGURE 2:**
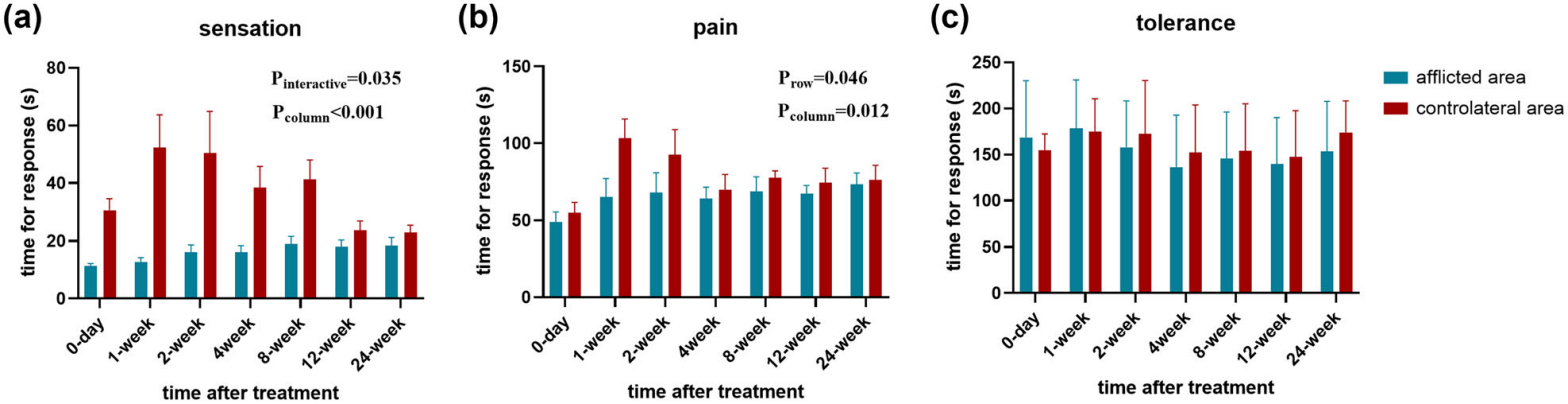
The electrical pain threshold assessed at each follow‐up. The column factor refers to time after treatment; the row factor refers to the place of the measured skin.

**FIGURE 3:**
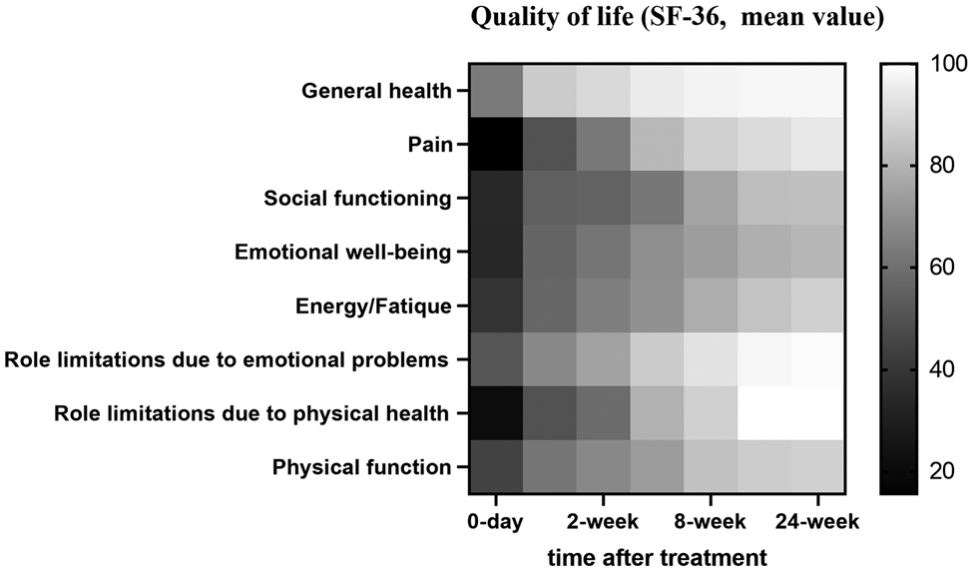
Heat map for the quality of life based on Short‐Form 36 scale.

For primary outcome, no PHN events occurred, with all patients report pain intensity ≤3 in the VAS scale in 3 months after treatment. Our results also present a significant time‐dependent pain relief phenomenon (*p* < .001, one‐way ANOVA, Figure 1), as the mean pain score before treatment is 7.0 ± 0.9, and decreases to 1.1 ± 0.8 in 6 months later. Besides, we have also analyzed the outcomes of pain threshold assessed at the affected area and the contralateral healthy skin. We find that the threshold for electrical sensation of the affected skin increase significantly after time and smoothly decreased thereafter (*p* = .035, two‐way ANOVA, Figure 2A). In the electrical pain threshold, we find a similar tendency in the affected skin (*p* = .046, two‐way ANOVA, Figure 2A). Interestingly, we also find out that the contralateral healthy skin experiences a mildly increase in both sensation and pain thresholds in 1–2 weeks after treatment (Figure 2A and B). No significance has been found in pain endurance time (Figure 2C). Other secondary outcomes are presented in Table 3 and Figure 3. According to our records, although no PHN occurred, one patient has experienced a deteriorated pain in short‐term follow‐ups. Besides, most of the symptoms of neuropathic pain, for instance, burning pain or evoke pain, disappears gradually, while several patients report remaining paresthesia in the healed skin area in 6 months. For the assessment of their life quality, the heat map presents a gradual but significant increase in all of the eight aspects in SF‐36 scale (*p* < .001, Figure 3). For consumption of analgesics, all patients withdraw tramadol in 1 week after injection, and the consumption of pregabalin decreases timely as well, with 4 of them finally withdraw pregabalin in 3 months later.

**TABLE 3:** Secondary outcomes

	Baseline	1 week	2 weeks	4 weeks	8 weeks	12 weeks	24 weeks
Pain intensity (No. of patients)							
Mild (VAS ≤3)	0	5	4	5	6	8	8
Moderate (VAS 3–5)	1	2	2	2	2	0	0
Severe (VAS > 5)	7	1	2	1	0	0	0

Likert scale (No. of patients)							
1–2	–	1	1	0	0	0	0
3	–	0	1	1	0	0	0
4–5	–	7	6	6	8	8	8

Neuropathic pain (No. of patients)							
Burning pain	–	8	5	4	2	0	0
Touching pain	–	8	7	2	2	2	0
Pressing pain	–	8	8	6	3	1	1
Evoked pain	–	7	2	2	0	0	0
Paresthesia	–	2	4	5	4	3	2

Quality of life (SF‐36, mean ± SD)*							
General health	63.1 ± 15.2	86.3 ± 13.4	90 ± 8.7	95 ± 7.5	96.9 ± 5	97.5 ± 5	97.5 ± 5
Pain	15.6 ± 14.8	50 ± 23.1	62.5 ± 20.6	81.3 ± 27.2	87.5 ± 25	90.6 ± 24.8	93.8 ± 16.5
Social functioning	34.3 ± 16.9	54.6 ± 24.6	55.4 ± 18.4	62.3 ± 15	75.3 ± 17.1	82.5 ± 13.3	83.3 ± 14
Emotional well‐being	33.6 ± 16.8	56.5 ± 15.9	61.5 ± 20	69 ± 16.3	73.8 ± 14	78.6 ± 9.7	80.5 ± 9.7
Energy/fatigue	38.8 ± 20.4	56.9 ± 17.8	64.4 ± 18.3	70 ± 14.8	78.1 ± 10.9	84.4 ± 6.3	87.5 ± 5.6
Role limitations due to emotional problems	51.3 ± 20.9	67.5 ± 21.1	75 ± 20.6	86.3 ± 14.9	92.5 ± 13	97.5 ± 6.6	98.8 ± 3.3
Role limitations due to physical health	20.9 ± 17.1	49.9 ± 23.4	58.4 ± 32.4	79.3 ± 33	87.5 ± 33.1	100 ± 0	100 ± 0
Physical function	44.5 ± 17.4	61.5 ± 19.7	67.5 ± 18	73 ± 17.7	83.5 ± 5.8	86.5 ± 5.6	87.5 ± 4.2

Analgesics (No. of patients)							
Pregabalin							
225 mg, 2/day	4	0	1	0	0	0	0
150 mg, 2/day	4	8	4	2	1	0	0
75 mg, 2/day	0	0	3	5	3	4	4
Withdraw	–	0	0	1	4	4	4
Tramadol	8	0	0	0	0	0	0

Likert scale: 1 = very unsatisfied, 2 = unsatisfied, 3 = neither satisfied nor unsatisfied, 4 = satisfied and 5 = very satisfied.

* *p* < .05 in each of the items in SF‐36 scale with the factor of time, using one‐way ANOVA.

## 4 DISCUSSION

In this trial, we identify that autologous fat grafting to the paravertebral space of the algogenic segment is a potentially valid method to prevent PHN. Among the eight patients accepted fat grafting, most of them have experienced immediate pain relief after the intervention, while eight of them have a lasting allogenic effect in all follow‐ups. No patients reported PHN in the 3‐month and longer follow‐ups, with a VAS score over 3 points. For other results, we identified that in accordance with the significant allogenic effect, autologous fat grafting also results in significant improvements in the mental state and the quality of life. Besides, for the eight patients with positive treatment response, the duration and consumption of analgesics is significantly reduced. In the assessment of pain thresholds, we identify that fat grafting differentially increases sensation and pain threshold in HZ area and healthy skin of patients. No serious adverse events occurred.

Autologous fat grafting for HZ is a new clinical use for this proven technique, yet evidence is confined. Previously, Sollie et al. (2020) first launched a clinical trial and proven that autologous fat grafting is a safe and feasible technique in alleviating neuropathic pain resulting from PHN, and the grafting area they choose is the dermal area with chronic pain. According to their theory, by grafting the fat to the affected skin could reduce neuroinflammation and restore the skin innervation. Yet this remains to be the only one report for fat grafting to treat PHN, with numerous questions, raged from basic mechanism to its clinical applications, to be solved. Several advantages or differences or advantages of our trial from Dr. Martin et al.’s study should be emphasized. First, the injection site in our trial is the parathoracic space, where the varicella‐zoster virus mainly colonized. Second, the amount of harvested fat is about 10–15 mL in each patient, while 200–300 mL of harvested fat is needed in the previous trial; this small amount of fat harvesting refers to minimally invasive. Third, the intervention time point of our trail is at the early stage of HZ to prevent PHN.

At present, the pathogenesis of PHN is not completely understood. The principle of treatment is a long‐term control of pain syndrome, relief of accompanied sleep and mood disorders, as well as improvement of life quality (Schlereth et al., 2015). Currently there have no standard treatment method, while several guidelines recommend pharmacal therapy and minimally invasive techniques. First‐line medications includes calcium channel blockers (such as gabapentin, pregabalin), tricyclic antidepressants, lidocaine patches, opioid analgesics, etc., while interventional therapy includes nerve interventional therapy (such as nerve block, selective nerve damage, intrathecal drug infusion therapy, etc.) and nerve regulation therapy (such as pulse radiofrequency therapy, nerve electrical stimulation technology, etc.) (Aysel et al., 2021; Kim et al., 2017). However, none of these treatments provides with reliable long‐term effect, and they often accompanied by various adverse events. Considering this, effective prevention methods for PHN form initiating are urgent.

The main theory for fat grafting to treat neuropathic pain is that the harvested fat tissue contains abundant of adipose tissue‐derived stem cells (ADSC), growth factors and anti‐inflammatory molecules, which accelerate the repairing damaged nerves, and further reliefs pain syndrome (Dehdashtian et al., 2020) . Recently, results of multiple studies in vitro support the notion that ADSC medication reduces allodynia associated with neuropathy and inhibits pain‐related behavior in chronic constriction injury (CCI) mouse, STZ‐diabetic mouse, and rat model of spinal cord injury while with no serious side effects (Hye et al., 2015). Besides, a variety of adipokines in fat, such as adiponectin, leptin, resistin, and endolipin may affect pain modulation (Gandhi et al., 2013; Walsh et al., 2016). Sun et al. (2018) have reported that adiponectin knockout mice had a lower pain threshold. Further studies have shown that adiponectin may modulate pain signals by inhibiting the activation of P38 MAPK, TRPV1, and CGRP in the dorsal root ganglion. Studies have also shown that fat tissue contains mesenchymal stem cells, which can promote nerve repair through differentiation into Schwann‐like cells and paracrine action (Ayad et al., 2015).

By review of literatures, we further proposed that the paravertebral space might be a better target location, as grafting fat directly affects the dorsal root ganglion. In animal studies, Shu‐Hung et al. (2015) showed that fat transplantation can reduce the levels of proinflammatory factors IL‐1β, COX‐2, iNOS, nNOS, and TNF‐α in dorsal root ganglion, and further reduce neuronal apoptosis by inhibiting AKT and JNK phosphorylation pathways in astrocytes. In line with these results, several clinical trials recommend that, besides from the common antivirus and analgesic medications, injection of solutions (containing dexamethasone and local anesthetics) to the diseased ganglion or spinal session is effective for HZ (Christo et al., 2007; Winnie & Hartwell, 1993), and the underlying mechanisms indicate that treatment targeted to the diseased DRG might be effective in preventing PHN from initiating, but rather alleviate pain form developed PHN.

Several limitations should be mentioned. First, the small sample size and a single‐armed design limits the interpretation of our results. Besides, we only investigate this treatment for HZ patients with a rash in chest and back, application of fat grafting intrathecally or to the peripheral nerve may also works. What is more, in this pilot trail, all participants are female, which may limit the explanation of our conclusions. In the further randomized control study, more patients with both genders will be included to test the safety as well as the effectiveness of this novel treatment for PHN.

## 5 CONCLUSION

In this prospective, single‐armed trial, we first report the feasibility and the safety of autologous fat transplantation to the paravertebral space in preventing PHN. More advantages of this technique including rich in fat content, easy to obtain, cost‐benefit as well as less adverse events makes it a promising treatment method in preventing PHN. The next step toward routine clinical translation is to perform a randomized, blinded, placebo‐controlled trial to investigate the actual long‐term benefice of autologous fat grafting to the paravertebral space in preventing PHN for early HZ.

## AUTHOR CONTRIBUTIONS

XJ Li designed the trial and conducted the experiments, R Tao conducted the experiments, XY meng analyzed the data and wrote the manuscript, H Wang revised the manuscript, HD Bi conceived the study and conducted the experiments, YC Xiong conceived the study. All authors read and approved the final version.

## CONFLICT OF INTEREST STATEMENT

The authors declare that they have no competing interests.

### PEER REVIEW

The peer review history for this article is available at https://publons.com/publon/10.1002/brb3.2918.

## Data Availability

Data are available from the corresponding author with reasonable requesting.
